# Field evolved insecticide resistance in the cotton mealybug *Phenacoccus solenopsis* and its direct and indirect impacts on the endoparasitoid *Aenasius arizonensi*s

**DOI:** 10.1038/s41598-022-20779-3

**Published:** 2022-10-06

**Authors:** Karuppan Shankarganesh, Michele Ricupero, Subramanian Sabtharishi

**Affiliations:** 1 ICAR-Central Institute for Cotton Research, Regional Station, Coimbatore, 641 003 India; 2 Department of Agriculture, Food and Environment, University of Catania, Via Santa Sofia, 100, 95123 Catania, Italy; 3 Dr. Subramanian Sabtharishi Division of Entomology, ICAR-Indian Agricultural Research Institute, New Delhi, 110 012 India

**Keywords:** Zoology, Entomology

## Abstract

*Phenacoccus solenopsis* Tinsley (Hemiptera: Pseudococcidae) an invasive mealybug on cotton is primarily controlled by conventional insecticides. An endoparasitoid *Aenasius arizonenesis* (Girault) (Hymenoptera: Encyrtidae) is a potential biocontrol agent of this pest. We assessed the susceptibility in field populations of *P. solenopsis* and *A. arizonensis* to commonly used insecticides: profenofos, imidacloprid and thiodicarb*.* Reproductive traits of the parasitoid and Environmental Risk Assessment (ERA) parameters viz., Reduction coefficient, Descriptive analysis, Risk Index (RI), Selectivity ratio and Hazard quotient were measured to assess the direct and indirect effects of these insecticides on the parasitoid. Probit analysis revealed heterogeneity in the insecticide resistance development for both the cotton mealybug and its parasitoid. The field populations of *P. solenopsis* exhibited resistance to profenofos (18.87–59.86 folds) and thiodicarb (20.07 folds) and susceptibility to imidacloprid. Development of resistance to profenofos was observed in field populations of *A. arizonensis*. Exposure to lethal doses of imidacloprid and profenofos caused a reduction in parasitization (19–23%) and adult emergence (62–69%) of the parasitoid. Profenofos, thiodicarb and imidacloprid were found to be hazardous, non-selective and harmful to the endoparasitoid, *A. arizonensis.* There is an urgent need for optimizing insecticide applications for sustainable management of this invasive mealybug in cotton.

## Introduction

The introduction of alien species and their establishment outside their native range dramatically concern agricultural ecosystems worldwide^1^. Mealybugs are invasive insect pests which can rapidly spread to new areas due to their cryptic behaviour, hostplant plasticity^2^ and high reproduction rate^3^. Cotton mealybug *Phenacoccus solenopsis* Tinsley (Hemiptera: Pseudococcidae) is one such pernicious pest that had emerged as a pest of cotton during the 1990s in the USA^4^. Over the last three decades, this mealybug pest invaded and established in > 43 countries in different parts of the world^5^. Its invasion has caused a severe economic loss in Ecuador, Chile, Argentina, Brazil, Pakistan, Nigeria, India, China, Egypt^6^ and Moracco^7^. Management of this mealybug pest has become difficult to manage owing to its invasive spread and impervious mealy coating to insecticides^8^. The immediate threat to cotton production posed by *P. solenopsis* in Asian countries has led to the intensive and irrational use of conventional insecticides for its management^9^. As a consequence, field populations of *P. solenopsis* have developed resistance to traditional and novel insecticides^9–11^.

Biological control represents a promising and sustainable approach for the management of *P. solenopsis* and it has to be prioritized^3^. Among the fortuitous natural enemies, the solitary endoparasitoid *Aenasius arizonensis* (Girault) (Hymenoptera: Encyrtidae) was considered a successful biocontrol agent of *P. solenopsis*^12^, because of its high parasitism rates recorded in the field^13^. This parasitoid, *A. arizonensis* has been used for implementing nationwide biological control programs to manage *P. solenopsis* in India, Pakistan and China^14–16^.

Around 600 arthropod species have been reported showing resistance to at least one pesticide^17^. An intriguing aspect of Arthropod Pesticide Resistance Database (APRD) 2022 is the growing number of cases of resistance in non-target arthropods, with 45 reported cases of pesticide resistance in parasitoids, predators and pollinators. Parasitoids seem to exhibit a higher susceptibility to pesticides compared to predators, as they are directly exposed to selection pressure. Parasitism may enhance the detoxification system in the host^18^. Therefore, insecticide resistance is more likely to evolve in parasitoids whose hosts have already developed considerable resistance to insecticides.

Besides insecticide resistance, pesticides pose a negative impact on non-target beneficial arthropods, which play a vital role in the ecosystem^19,20^. The risk assessment of insecticides is basically required in the integrated pest management (IPM) context because their irrational use can cause serious consequences on the ecological services offered by non-target beneficial arthropods^21,22^. The destruction of natural enemies can exacerbate pest problems as they play an important role in regulating pest population levels. Annihilation of natural enemies in cropping systems would lead to an adverse scenario of the use of a higher dose of toxicants leaving the enhanced residue of hazardous toxicants in the environment^23^. Additionally, pesticides can also affect life-history parameters including growth rate, development time, reproductive functions and the preying/parasitization potential of natural enemies^24^.

Regulations are in place to assess the non-target effects of insecticides and safety standards have been enacted as per Document on Terrestrial Ecotoxicology (SANCO/10329/2002, 2002), SETAC/ESCORT Guidance Document^25,26^ and IOBC guidelines on classification of insecticides based on their non-target effects on natural enemies in agricultural eco system^27^. The safety standards imposed by regulatory agencies are often challenged by the abuse of insecticides when invasive pests are spread in epidemic proportions as in the case of cotton mealybug *P. solenopsis*. Limited literature is available on the susceptibility levels of field populations of the cotton mealybug and its parasitoids to insecticides in India^9^. Similarly, eco-toxicological risk assessment of insecticides in field populations of *A. arizonensis* has scarcely been documented^28^.

The field populations of the pest and parasitoid were collected from four major cotton-growing regions across India. The choice of insecticides was done based on the inputs from a Knowledge Attitude Practice survey^29,30^ conducted in the field locations. Detailed log dose probit analyses were done to ascertain the susceptibility levels of the pest and parasitoid to the contemporarily used insecticides in cotton. The indirect effect of insecticides on the parasitization potential of *A. arizonensis* was assessed through estimation of Environmental Risk Assessment (ERA) parameters such as Reduction Coefficient (Ex), Descriptive analysis (E), Risk Index, Selectivity ratio and Hazard quotient^20,27,31^. Understanding susceptibility levels of the field populations of the pest and assessing the target and non-target impacts of insecticides on its potential biocontrol agent would help to optimize the strategies for sustainable management of this invasive cotton mealybug.

## Results

### Insecticide usage history and cropping details

Details of the Knowledge-Attitude-Practice surveys are presented in Table 1. The surveys revealed that the commercial *Bt* cotton hybrid seeds available to the farmers had been pre-treated with imidacloprid 70WS. The mealybugs were the predominant sucking pests not only on cotton but also on other vegetable crops in the survey areas, while, whitefly, *Bemisia tabaci* (Genn.) (Hemiptera: Aleyrodidae), and the leafhopper, *Amrasca biguttula biguttula* Ishida (Hemiptera: Cicadellidae) were other sucking pests noticed on cotton. The OPs, carbamates, pyrethroids, and neonicotinoids are the predominant group of insecticides being used by the farmers to control mealybugs and other sucking pests on cotton. The number of spray applications was 10–12 in the Ludhiana and Saoner; 8–10 sprays in Junagadh and Chhindwara locations of India.

**Table 1:** Detailed information on Indian populations of *Phenacoccus solenopsis* used in the current study.

Name of the population	Geographic origin (agro-climatic zone—States)	GPS coordinates	Common insecticides used for controlling sucking pest of cotton	Average number of spray by cotton farmers	Stage of the crop	Adjacent crops	Remark (observed pest and practice)
Junagadh, (Gujarat, India)	South Saurashtra Agro Climatic Zone	21°48′ 43″ N 70° 44′ 05″ E	Imidacloprid, Profenofos, Acetamipirid, Fipronil, Monocrotophos, Thiodicarb Dimethoate, Buprofezin, Chlorpyrifos, Cypermethrin,	8–10	Flowering	Pigeon pea	Mealybug, Whitefly, Jassids, Aphids, Red cotton bug. Pre treatment of seed with imidacloprid; 8 rounds of sprays against sucking pests
Ludhiana (Punjab, India),	Trans Gangetic Plains Region-Punjab	30° 53′46″ N 75° 51′ 32″ E	Thiodicarb, Buprofezin, Dimethoate, Chlorpyrifos, Profenofos, Monocrotophos, Cypermethrin, Deltamethrin	10–12	Boll busting	Cotton and Okra	Mealybug, Whitefly, Jassids, Aphids, *Earias* sp. Pre-treatment of seed with imidacloprid; 10–12 rounds of sprays against sucking pests; 2- 3 sprays for mealybug control
Saoner (Maharashtra, India),	Central Vidarbha	21° 38′ 59″ N 78° 92′ 14″ E	Imidacloprid, Profenofos, Dimethoate, Buprofezin, Chlorpyrifos, Fipronil, Monocrotophos, Cypermethrin, Deltamethrin, lambdacyhalothrin	10–12	Boll formation	Sorghum	Mealybug, Whitefly, Jassids, Aphids, Red cotton bug. Pre-treatment of seed with imidacloprid; 2–4 rounds of sprays with imidacloprid followed by other chemicals
Chhindwara (Madhya Pradesh, India)	Satpura Plateau	22° 07′ 56″ N 78° 93′ 30″ E	Imidacloprid, Thiodicarb Buprofezin, Chlorpyrifos, Monocrotophos	8–10	Boll Formation	Sorghum	Mealybug, Whitefly, Jassids, Aphids, Red cotton bug. Pre-treatment of seed with imidacloprid; 8–10 sprays targeted against sucking pests

### Acute toxicity of insecticides on *P. solenopsis*

According to the probit model, there were no significant differences between the observed and the expected data, validating thus the estimated lethal concentrations for the tested chemicals. The variation in susceptibility of *P. solenopsis* to imidacloprid, profenofos and thiodicarb was noticed between the four field populations (Table 2). The mealybug field populations were the least susceptible to profenofos with the LC_50_ values being in the range of 27.74 mg L^−1^ (χ^2^ = 0.782, (df) = 5, P = 0.941) (Ludhiana) to 88.00 mg L^−1^ (χ^2^ = 0.429, (df) = 5, P = 0.980) (Saoner). When compared to laboratory susceptible check, *P. solenopsis* field populations were found to be 18.87–59.86 folds resistant to profenofos. Significant differences in susceptibility to thiodicarb were observed with the LC_50_values ranging from 5.643 mg L^−1^ (Saoner) to 52.88 mg L^−1^ (Ludhiana) and the field populations of *P. solenopsis* were showing up to 20.07 folds resistance to thiodicarb. Comparatively, imidacloprid was found to be relatively more toxic to *P. solenopsis;* the field populations were showing just 1.67–8.79 folds resistance to the neonicotinoid compound in comparison to the susceptible check.

**Table 2:** Log-dose probit estimated data of imidacloprid, profenofos and thiodicarb against field populations of *Phenacoccus solenopsis*.

Insecticide	Population	n	Slope ± SE	χ^2^ (df)	*P*	LC_50_ (mg L^−1^)	FL 95%	RR	LC_99_ (mg L^−1^)	FL 95%
Imidacloprid	Junagadh	210	0.804 ± 0.125	3.284 (5)	0.511	16.89	8.996–30.269	5.78	224.42	126.95–596.10
	Ludhiana	210	0.462 ± 0.114	0.553 (5)	0.968	4.87	0.816–12.855	1.67	127.61	63.01–416.26
	Saoner	203	0.798 ± 0.126	6.445 (5)	0.168	25.68	7.475–90.063	8.79	348.76	157.65–868.36
	Chhindwara	210	0.811 ± 0.126	4.468 (5)	0.346	23.34	4.289–117.15	7.99	408.35	207.42–946.48
	Lab population	232	0.792 ± 0.194	0.922 (5)	0.038	2.92	2.333–3.672	1.00	56.132	31.24–139.05
Profenofos	Junagadh	220	0.747 ± 0.129	5.009 (5)	0.287	82.23	33.057–65.14	55.94	506.10	278.51–974.16
	Ludhiana	232	0.785 ± 0.124	0.782 (5)	0.941	27.74	5.519–55.21	18.87	405.22	221.43–737.91
	Saoner	210	0.769 ± 0.128	0.429 (5)	0.980	88.00	24.730–20.57	59.86	525.60	267.76–805.05
	Chhindwara	240	0.693 ± 0.122	1.978 (5)	0.740	54.76	22.842–12.61	37.25	694.79	219.78–869. 26
	Lab population	240	1.294 ± 0.275	0.392 (5)	0.096	1.47	1.011–2.091	1.00	24.92	10.55–51.65
Thiodicarb	Junagadh	210	1.304 ± 0.163	2.131 (5)	0.712	16.75	11.041–5.116	6.36	616.25	242.12–1155.30
	Ludhiana	222	0.785 ± 0.124	0.280 (5)	0.991	52.88	39.92–110.46	20.07	421.79	205.22–817.72
	Saoner	242	0.619 ± 0.119	2.861 (5)	0.582	5.643	1.832–11.926	2.14	89.57	49.35–241.59
	Chhindwara	210	0.699 ± 0.121	2.946 (5)	0.567	35.38	18.341–53.874	13.43	558.85	259.91–802.63
	Lab population	232	0.676 ± 0.221	1.467 (5)	0.367	2.635	2.038–3.371	1.00	44.412	35.56–158.46

n, number of insects tested; SE, Standard Error; χ^2^, chi-square testing goodness of fit of concentration-mortality response; df, Degrees of freedom; LC in mg L^−1^, Lethal Concentration; FL, Fiducial Limits; Relative Resistance (RR) = LC_50_ of field collected test population/LC_50_ of Laboratory Susceptible check.

### Residual toxicity of insecticides on *A*. *arizonensis*

The Probit dose-response mortality assays revealed that profenofos was relatively more toxic to all the field populations of mealybug endoparasitoid, *A. arizonensis.* The LC_50_ values were ranging from 0.0009 mg L^−1^ (χ^2^ = 4.432, (df) = 5, P = 0.490) in Chhindwara to 0.0060 mg ai L^−1^ (χ^2^ = 10.32, (df) = 5, P = 0.066) in Junagadh population (Table 3). Imidacloprid was found to show the least residual toxicity against *A. arizonensis* with the LC_50_ values being significantly lower: 0.0010 mg L^−1^ (Chhindwara) to 0.0045 mg L^−1^ (Ludhiana). Next to profenofos, thiodicarb also had high residual toxicity to all the field populations of *A. arizonensis* as shown by the LC_50_ values in the range of 0.0018–0.0043 mg L^−1^. The field populations of *A. arizonensis* were showing 9–60 folds resistance to profenofos; 5–22.5 folds resistance to imidacloprid compared to the lab susceptible check. Relatively less resistance to thiodicarb (RR in the range of 4.28–10.24) was observed in the field populations of *A. arizonensis* populations.

**Table 3:** Residual toxicity of imidacloprid, profenofos and thiodicarb against field populations *Aenasius arizonensis*.

Insecticides	Populations	Slope ± SEm	χ^a^ (df)	*P*	LC_50_ (mg L^−1^)	Fiducial limit 95% CI		RR
						Lower	Upper	
Imidacloprid	Junagadh	1.750 ± 0.270	4.830 (5)	0.437	0.0036	0.0030	0.0042	18.0
	Ludhiana	2.450 ± 0.341	10.33 (5)	0.066	0.0045	0.0022	0.0083	22.50
	Saoner	2.127 ± 0.314	4.957 (5)	0.421	0.0035	0.0220	0.0460	17.50
	Chhindwara	1.136 ± 0.162	7.310 (5)	0.198	0.0010	0.0008	0.0014	5.00
	Lab population	0.798 ± 0.197	0.906 (5)	0.038	0.0002	0.0001	0.0008	1.00
Profenofos	Junagadh	1.860 ± 0.314	10.32 (5)	0.066	0.0060	0.0046	0.0079	60.0
	Ludhiana	1.595 ± 0.254	12.03 (5)	0.034	0.0036	0.0026	0.0046	36.0
	Saoner	3.255 ± 0.468	4.652 (5)	0.460	0.0031	0.0024	0.0042	31.0
	Chhindwara	1.300 ± 0.186	4.432 (5)	0.490	0.0009	0.00021	0.00072	9.00
	Lab population	1.294 ± 0.275	0.392 (5)	0.075	0.0001	0.00008	0.00015	1.00
Thiodicarb	Junagadh	1.931 ± 0.289	5.166 (5)	0.396	0.0030	0.0020	0.0050	7.14
	Ludhiana	1.660 ± 0.256	7.545 (5)	0.183	0.0030	0.0010	0.0080	7.14
	Saoner	2.301 ± 0.326	6.909 (5)	0.227	0.0043	0.0024	0.0062	10.24
	Chhindwara	1.106 ± 0.183	5.370 (5)	0.372	0.0018	0.0012	0.0032	4.28
	Lab population	1.351 ± 0.222	1.065 (5)	0.056	0.00042	0.00024	0.00085	1.00

SE, Standard Error; χ^2^,chi-square test of goodness of fit of concentration-mortality response; df, Degrees of freedom; LC, Lethal Concentration; FL, Fiducial Limits; Relative Resistance (RR) = LC_50_ of field population/LC_50_ of laboratory reared Susceptible check.

### Indirect effect of insecticides on parasitoids

**Figure 1:**
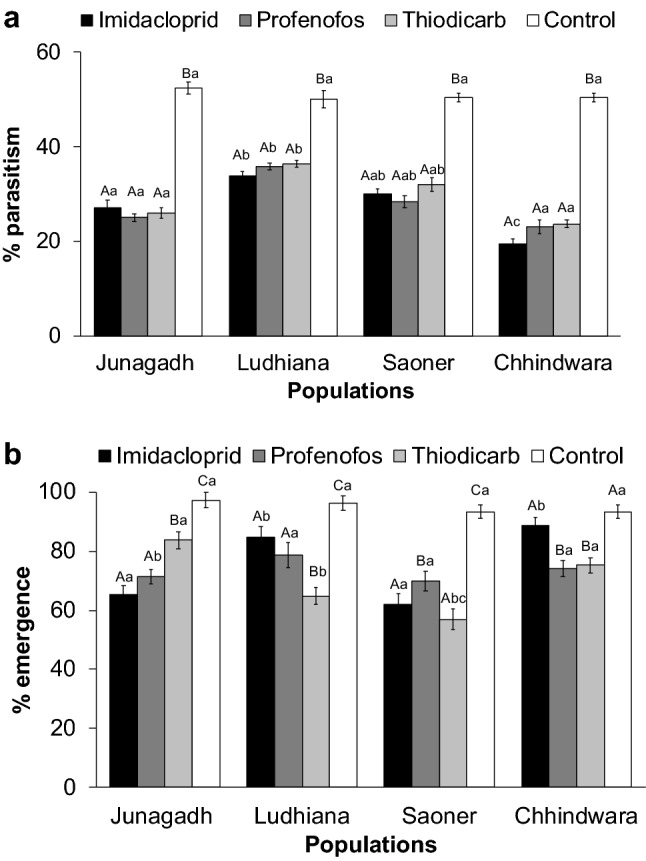
Effect of insecticides on the parasitism (%) emergence rate in field populations of mealybug parasitoid, *A.arizonensis.* Mean ± SE parasitism rate (**a**) and progeny emergence rate (**b**) of *Aenasius arizonensis* females exposed by contact residue of LC50 of imidacloprid, profenofos and thiodicarb. Significant effect of the factors insecticides, populations and their interactions on both the parasitism rate and the emergence rate. All the tested insecticides negatively affected the parasitism and the emergence. Columns bearing the same letter (upper case letters: within the same population; lower case letters: within the same tested insecticide) are not significantly different (LSD post hoc test for multiple comparisons at P ≥ 0.05).

While the direct effect of insecticides on the parasitoid was revealed by the dose-response assays, the indirect effects of insecticides were assessed through estimation of parasitization potential and adult emergence of *A. arizonensis.* Laboratory assays have shown that the insecticidal residues (tested @ LC_50_ concentrations of the respective insecticides for the respective field population) significantly affected the parasitism and the emergence rates of *A. arizonensis* (Fig. 1). The statistical analysis revealed a significant effect of the factors insecticide, populations and their interaction on both the parasitism rate (F3, 80 = 362.44; P < 0.0001; F3, 80 = 50.13; P < 0.0001; F3, 80 = 8.08; P < 0.0001) and the emergence rate (F3, 80 = 410.91; P < 0.0001; F3, 80 = 97.12; P < 0.0001; F3, 80 = 59.09; P < 0.0001). All the tested insecticides negatively affected the parasitism and the emergence rates of *A. arizonensis* in comparison to the Lab population. In particular, imidacloprid significantly decreased the parasitism rate to the tune of to 19.4 ± 1.1% in *A. arizonensis* collected from Chhindwara location.

### Environmental risk assessment

The environmental risk assessment parameters such as Descriptive analysis (E), Reduction Coefficient (Ex), Risk Index (RI), Selectivity Ratio (SR) and Hazard quotient (HQ) were computed for categorizing the relative safety of the insecticides to the mealybug parasitoid *A. arizonensis* (Tables 4 and 5). Descriptive analysis (E) revealed that all the three insecticides tested were slightly toxic to *A. arizonensis* populations except for thiodicarb in Ludhiana region (E = 27.68). The Reduction Coefficient (Ex) values (60.84–82.77%) revealed that all the test insecticides were slightly harmful to most of the field populations of *A. arizonensis* (Table 5). The Risk indices (RI) were ranging from 0.04 to 0.75 in the field populations of *A. arizonensis* and based on the RI values, imidacloprid and thiodicarb could be categorized as low risk compounds (RI < 0.5). Conversely, profenofos posed a high risk to the parasitoid with the RI > 0.5 for all the locations. A Perusal of the selectivity ratio revealed that all the three insecticides were non selective to *A. arizonensis* with the selectivity ratio being less than 1 for the field populations of *A. arizonensis* tested. The HQ values suggest that the imidacloprid was relatively safe to the parasitoid collected from Junagadh, Ludhiana and Saoner locations.

**Table 4:** Descriptive analysis of reduction in parasitism and emergence rates of *Aenasius arizonensis* females exposed to lethal doses of the insecticides.

Population	Imidacloprid				Profenofos				Thiodicarb			
	E (A)	I	E (B)	I	E (A)	I	E (B)	I	E (A)	I	E (B)	I
Junagadh	48.47	2	65.49	2	79.78	2	64.71	2	48.74	2	57.65	2
Ludhiana	35.50	2	43.92	2	68.31	2	44.71	2	27.68	1	53.73	2
Saoner	42.75	2	63.53	2	77.75	2	61.18	2	36.42	2	64.31	2
Chhindwara	62.98	2	66.27	2	81.12	3	67.06	2	52.98	2	65.10	2

Descriptive analysis (E) = Reduction in parasitism (A) or emergence (B) in percent and their respective IOBC toxicity classes (I). Parasitization and emergence rates were estimated for the females of *A.arizonensis* (of respective field populations) exposed to lethal doses (LC_50_) of imidacloprid, profenofos and thiodicarb.

E(A) = Per cent reduction in parasitization (%); E(B) denotes percent reduction in emergence (%) I refers to IOBC Toxicity classes: 1 = harmless, 2 = slightly harmful, 3 = moderately harmful, 4 = harmful.

**Table 5:** Environmental Risk Assessment (ERA) parameters estimated for field populations of *Aenasius arizonensis*.

Insecticide	Population	Recommended dose (g ai ha^−1^)	E_x_	IOBC classification	Risk index	Risk category	Selectivity ratio (SR)	Hazard quotient (HQ)	Hazard category
Imidacloprid	Junagadh	25	74.83	Slightly harmful	0.31	Low risk	0.00021	27.778	Safe
	Ludhiana	25	68.30	Slightly harmful	0.14	Low risk	0.00092	22.222	Safe
	Saoner	25	61.46	Slightly harmful	0.24	Low risk	0.00014	28.571	Safe
	Chhindwara	25	82.77	Moderately harmful	0.51	Medium risk	0.00004	100	Slightly to moderately
Profenofos	Junagadh	500	81.15	Moderately harmful	0.73	High risk	0.00007	333.33	Slightly to moderately
	Ludhiana	500	71.81	Slightly harmful	0.58	Medium risk	0.00013	555.56	Slightly to moderately
	Saoner	500	72.66	Slightly harmful	0.70	High risk	0.00004	645.16	Slightly to moderately
	Chhindwara	500	78.69	Slightly harmful	0.75	High risk	0.00002	2222.2	Slightly to moderately
Thiodicarb	Junagadh	750	79.86	Slightly harmful	0.32	Low risk	0.00018	500	Slightly to moderately
	Ludhiana	750	60.84	Slightly harmful	0.04	Low risk	0.00006	500	Slightly to moderately
	Saoner	750	61.68	Slightly harmful	0.15	Low risk	0.00076	348.84	Slightly to moderately
	Chhindwara	750	77.79	Slightly harmful	0.37	Low risk	0.00005	833.33	Slightly to moderately

ERA parameters were estimated on the females of *Aenasius arizonensis* exposed to lethal doses of imidacloprid, profenofos and thiodicarb.

E_x_ denotes Reduction coefficient.

IOBC classification is based on Reduction coefficient values.

Risk category: categorizing of insecticides based on Risk Indices in 0–1 scale with 0 = safe and 1 = Highly risk.

Selectivity ratio (SR)values < 1 indicates the non selective nature of the insecticides;

Hazard category : If HQ is less than 50—a pesticide is considered safe, 50–2500—slightly to moderately toxic and > 2500 as dangerous.

## Discussion

The cotton mealybug *P. solenopsis* which has entered India in 2006 as an invasive pest^32^ continues to be a regular pest on cotton and horticultural crops^33^ owing to its pronounced polyphagia. The rapid outbreak of this invasive pest and inadequate control offered by chemical insecticides necessitated the exploration of biological control options in Asian countries. Large-scale release of solitary endoparasitoid *A. arizonensis* was explored in India, Pakistan and China^14–16^. The crops like cotton and vegetables (wherein *P. solenopsis* is a serious pest) receive frequent applications of similar insecticides for the control of sucking pests. The abuse of insecticides might have compromised the efficacy of both the chemicals and biological control strategies in managing the invasive mealybug *P. solenopsis* in India and Pakistan^34^. Studies has shown widespread development of resistance to different insecticides in *P. solenopsis* in India^9^ and Pakistan^35,36^.

Results of our present study have demonstrated varying levels of resistance to imidacloprid, profenofos and thiodicarb in four *P. solenopsis* populations in India. Interestingly, we recorded for the first time, the development of insecticide resistance in four different field populations of *A. arizonensis*, one of the most effective parasitoids recorded against the cotton mealybug.

The insecticide exposure–response relationship revealed significant variations in the susceptibility of *P. solenopsis* field populations to three insecticides belonging to organophosphates, carbamates and neonicotinoids. Preliminary surveys indicated that these insecticides were widely and repeatedly applied by farmers in the surveyed locations. The high range of LC_50_ values recorded in *P. solenopsis* field populations against profenofos reveals that this OP compound can no longer effectively control *P. solenopsis* under field conditions. Profenofos has been one of the widely used insecticides by Indian farmers for decades for the control of bollworms, whiteflies and mealybugs in the cotton system^37^. Worldwide, newer classes of insecticides have replaced organophosphates and carbamates for the control of sucking pests. However, these conventional insecticides are still under use in India, because they are less expensive^38^. The results of our survey in major cotton-growing regions in India have also proved this point. High resistance development to profenofos in *P. solenopsis* had been documented earlier in the Punjab province of Pakistan^35^. Similarly, low to moderate resistance to profenofos in field populations of *P. solenopsis* was reported in cotton-growing districts of Maharashtra in India^9^. Also, thiodicarb and imidacloprid showed reduced toxicity to *P. solenopsis* in two field populations of mealybug, *P. solenopsis* (Table 2).

The *Bt* cotton varieties were introduced in India during the early 2000s and presently about 96% of cotton cropped area in this country grows transgenic *Bt* cotton varieties. To manage the surge in the attack of sucking pests on *Bt* cotton, there has been persistent use of imidacloprid (as all *Bt* cotton seeds are mandatorily treated with imidacloprid). The continuous use of imidacloprid has predisposed the resistance development against imidacloprid in several cotton pests in India^30^. Similar to the results of our study, loss in toxicity to imidacloprid against *P. solenopsis* has earlier been reported in Pakistan and India^9,35^.

Insecticides often cause a deleterious impact on insect natural enemies in agricultural systems, although they are applied for controlling target pests. Adult parasitoids are more susceptible than their preimaginal stages to encounter insecticide by contact on spray drift or by ingestion of contaminated food (e.g., nectar and/or pollen) after insecticide application on the plant surfaces. The toxic residues on plant surface directly affect the survival of released parasitoids intended for controlling the target pests. A study by Nidheesh et al.^39^ has shown that profenofos and a neonicotinoid, thiamethoxam were found to be highly toxic to mealybug parasitoid, *A. arizonensis*.

The residual toxicity assays in the present study revealed that profenofos was highly toxic to *A. arizonensis* field populations. The parasitoid collected from Junagadh region was 60 times (*P* = 0.066) more resistant to profenofos. Field populations of *A. arizonensis* were found to be 7–10 folds more tolerant to thiodicarb as compared to the laboratory population. However, imidacloprid was found to show 5–22 folds reduced toxicity to the field populations of *A. arizonensis* when compared with susceptible check. The variability of insecticide susceptibility in field populations of *P. solenopsis* and *A. arizonensis* could be attributed to the differential levels of insecticidal pressure experienced by the pest and its parasitoid.

The slopes of the regression lines of probit analysis can provide clues on the efficacy of insecticides on the target insect population. In this study, the slopes of dose–response probit curves were extremely low (< 2.0) for mealybug, and its parasitoid, suggesting the heterogeneity in resistance development against the tested insecticides in both the mealybug pest and its parasitoid *A. arizonensis.* Results of our study indicate that the cotton mealybug is in an early stage of developing field-evolved resistance to insecticides and there is a concomitant increase in tolerance in field populations of *A. arizonensis* to insecticides such as profenofos.

Resistance development in cotton mealybug to insecticides is not surprising, considering the over-reliance on conventional chemical insecticides to contain the epidemic outbreak of cotton mealybug, the *P. solenopsis* in India and its neighbouring countries during the 2000s the and persistent use of chemicals for controlling other sucking pests on cotton^30^. The first instance of *P. solenopsis* population showing resistance to acetamiprid was documented in Pakistan^40^. Presently, this pest has developed resistance against 24 insecticides and there are 196 reported cases of insecticide resistance^41^. Under these circumstances, there is a definite likelihood of control failures of applied insecticides against the invasive mealybug, *P. solenopsis*.

Studies have shown that the application of insecticides such as profenofos significantly impairs the activities of parasitoids and predators like *A. arizonensis (*= *bambawalei)*, *Brumus suturalis* Fabricius (Coleoptera: Coccinellidae) and *Scymnus coccivora* Ayyar. (Coleoptera: Coccinellidae)^42^. Nalini and Manickavasagam^43^ reported the toxicity of profenofos and imidacloprid on *A. arizonensis.* Meenu and Ram^44^ had also observed that profenofos and thiodicarb resulted in maximum mortality of *A. arizonensis*. Our results suggest that thiodicarb is more toxic to the mealybug *P. solenopsis* but is relatively safer to its parasitoid *A. arizonensis*.

Besides the direct impact on parasitoids, the application of these insecticides significantly impacted their parasitization efficiency. Residual toxicity assays in this study showed that exposure to a lethal dose (LC_50_) of imidacloprid and profenofos caused a 19–23% reduction in parasitization and a 62–69% reduction in adult emergence. Earlier studies have shown that exposure of mummified mealybugs to insecticides like profenofos caused a deleterious effect on adult emergence, while, imidacloprid affected the fitness traits of *A. arizonensis*^28^.

Beneficial insects including natural enemies and pollinators constituted less than 3% of the total recorded cases of insecticide resistance in the 1980s. However, by 2015, the reported cases of insecticide resistance in natural enemies have risen to 6.4%^45^. According to the Arthropod Pesticide Resistance Database (APRD)^17^, there are about 45 cases of insecticide resistance reported in 18 species of hymenopterans against different groups of insecticides^41^. Ingestion of toxicants from the host mainly contributed to the development of insecticide resistance in endoparasitoids^46^. Insecticide resistance development in the host insect influenced the selection pressure in the hosted parasitoid^18^. Parasitoids associated with the resistant population of diamondback moth *Plutella xylostella* Linnaeus) (Lepidoptera: Plutellidae) also showed resistance to the same insecticides^47^. Thus, parasitoids too develop resistance to the insecticides in the long run, when the host harbouring it, is continuously exposed to the selection pressure from insecticides.

A comprehensive ecotoxicological risk assessment is needed to better understand the adverse impact of insecticides on non-target organisms. Regulations are in place to assess the non-target effects of insecticides and safety standards have been evolved like Document on Terrestrial Ecotoxicology^26^ and IOBC guidelines on the classification of insecticides based on their non-target effects on natural enemies in an agricultural ecosystem^27^.

Insecticide exposures severely affect the fitness traits of natural enemies^48,49^. There are limited studies on mortality assessment of insecticides on field populations of *P. solenopsis* together with its parasitoid^35,50,51^. The present study assessed the mortality in field populations of the cotton mealybug and its parasitoid and the insecticide effects on the reproductive traits of the endoparasitoid, *A. arizonensis*. Quantitative estimates such as Reduction Coefficient (Ex) and Descriptive analysis (E), selectivity ratio, Risk index and Hazard quotient have considered both the mortality of parasitoids and their parasitization efficiency to estimate the hazardous effect of pesticides on natural enemies^27,31^. Comparative assessment of Reduction Coefficient (Ex) revealed that the three commonly used insecticides (profenofos, imidacloprid and thiodicarb) for controlling *P. solenopsis* in the major cotton-growing regions could be considered slightly toxic to *A. arizonensis* according to the IOBC classification. The deleterious effect of these three insecticides has earlier been documented by Badshah et al.^50^. Imidacloprid was found to be slight to moderately toxic to the adults of *A. arizonensis* based on the assessment of Reduction Coefficient (Ex) as per an earlier report^28^. The harmfulness of profenofos and thiodicarb against *A. arizonensis* has well been documented earlier^44,51^. The results of the descriptive analysis revealed that, except for thiodicarb application in the Ludhiana population, all three insecticides were found to be slightly toxic to *A. arizonensis* in the study locations. Pazini, et al.^49^ classified imidacloprid as slightly harmful to *Telenomus podisi* Ashmead (Hymenoptera: Scelionidae) based on the assessment of Descriptive analysis.

The risk indices were found to be ranging from 0.04 to 0.73 for different field populations of the pest and parasitoid. Comparatively lower Risk Index suggests that application of thiodicarb and imidacloprid pose a relatively low risk to parasitization by the endoparasitoid. The OP compound, profenofos poses the highest risk to the parasitization by the endoparasitoid.

The selectivity ratio reveals that all the three insecticides were non-selective to the mealybug pest. Further studies are needed to identify insecticidal molecules or formulations selective to the cotton mealybug with the least non-target effect on its fortuitous parasitoid *A. arizonensis.*

**Figure 2:**
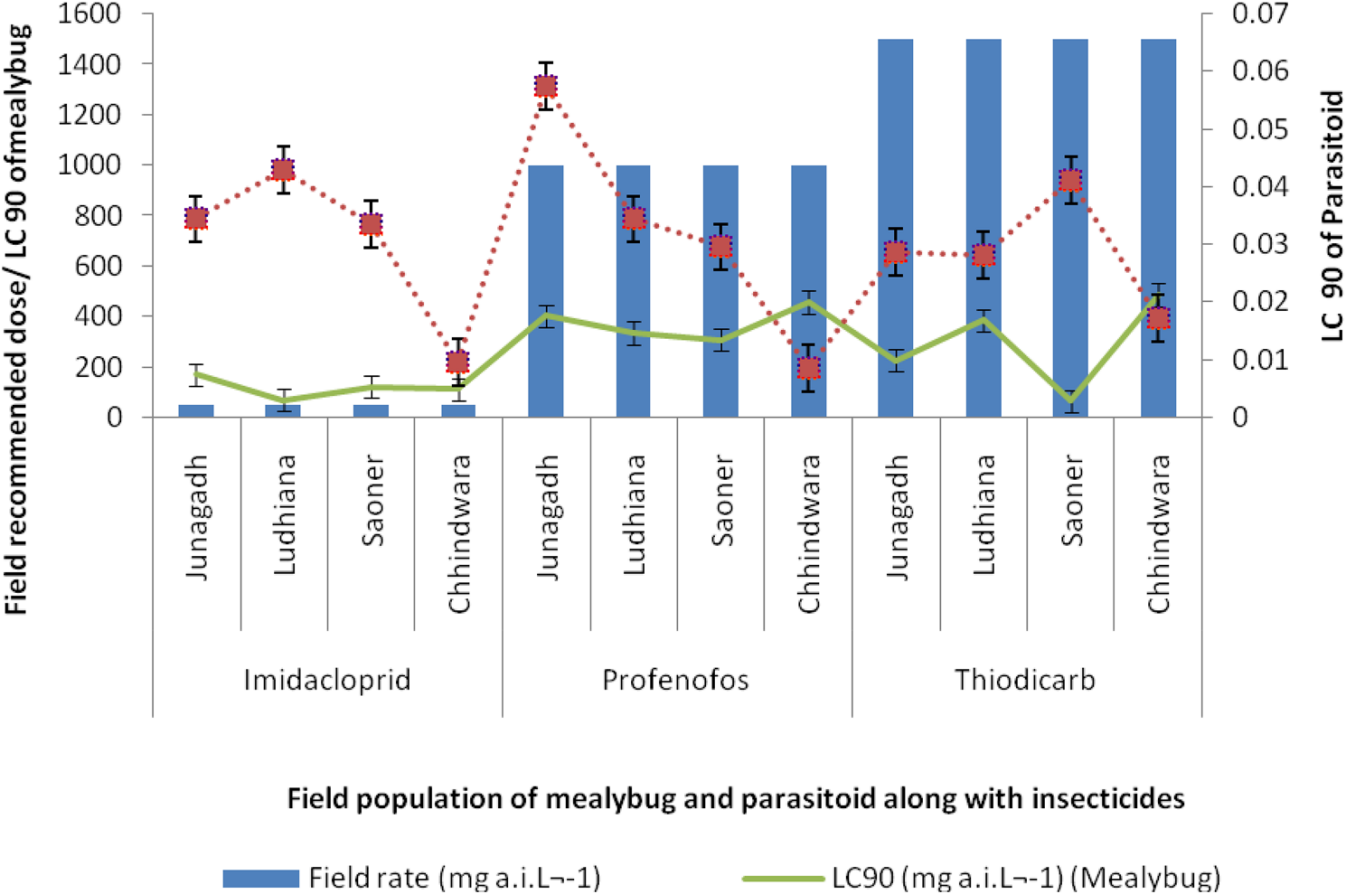
Comparison of contact LC90 values of insecticides to mealybug and parasitoid with their field recommended concentrations. The expected mortality of field populations of *P. solenopsis* as achieved by label rates is represented graphically. The toxic impact of these insecticides on *A. arizonensis* field populations could be deduced from this graph. Susceptibility of the four field populations of *P. solenopsis* as given by the estimates of LC_50_ and 95% confidence limits for the tested insecticides was compared with the maximum recommended field dose of these insecticides by Central Insecticides Board and Registration Committee (CIBRC), Government of India. As per CIBRC, the recommended doses for the tested insecticides against sucking pests were: imidacloprid 0.00625 mg L^−1^; profenofos: 0.125 mg L^−1^; thiodicarb: 0.185 mg L^−1^.

A hazard ratio HQ < 50 for a pesticide is considered safe for natural enemies of a pest. The HQ values recorded in this study suggest that the imidacloprid is relatively safe to the field populations of *A. arizonensis*. Even though HQ values suggest profenofos and thiodicarb are slight to moderately toxic to the parasitoid, their application at recommended label rates would likely to cause 90% mortality of *A. arizonensis* (Fig. 2). The vulnerability of this endoparasitoid the field recommended dose of profenofos and thiodicarb has earlier been documented^28^.

Currently, as many as 36 insecticides have been approved for use against sucking pest complex in cotton and other crops by the Central Insecticide Board of Registration Committee in India^52^. Our study has provided new knowledge on the direct and indirect impact of currently used insecticides on contemporary field populations of *P. solenopsis* and its main parasitoid in India. Both the cotton mealybug and its parasitoid are at the early stage of development of field resistance to insecticides. By deploying elaborate ERA parameters, our study has demonstrated that profenofos, thiodicarb and imidacloprid being widely used by cotton farmers are hazardous, non-selective and harmful to the potential biocontrol agent of mealybugs in the cotton ecosystem.

Detailed studies regarding the side effect of pesticides should be addressed towards the behavior of beneficial arthropods^53^ as well as the combination of pesticides with other stressors (e.g., temperature)^20^. There is a need for advocating the replacement of widely used insecticides belonging to OP and carbamates with newer chemistries for integrated management of the invasive mealybug, *P. solenopsis* in India and Pakistan.

Our studies reiterate the need for optimizing the insecticide usage for mitigating the insecticide resistance development in mealybug and conserving the fortuitous parasitoid, *A. arizonensis* in the cotton ecosystem. Adoption of biorational approaches involving botanical insecticides such as Neem pesticides, use of entomopathogens like *Metarhizium anisopliae, Beauveria bassiana, Lecanicillium lecanii,* and identifying safer and selective insecticidal molecules with the least non-target effect to natural enemies would ensure the sustainable management of *P. solenopsis* and other sap-sucking pests in the cotton crop.

## Methods

### Guidelines

All methods were performed following the relevant guidelines and regulations approved by the institution and funding agency. All experimental protocols were as per the technical programme of the research project as approved by the Institute Research Council ethics committee of the ICAR- Indian Agricultural Research Institute, New Delhi. Since the survey was interview-based with humans, before conducting the survey, we informed the farmers about the purpose and the utilization of the survey; an informed consent was obtained from each of the participants.

### Collection of insects, maintenance and rearing

No specific permissions were required for these locations/activities as the plant/insect species covered in this study are not endangered or protected species. The pest infestation was seen in natural conditions at different locations and their collection does not require any permission from any regulatory authority under the prevalent laws.

Field populations of cotton mealybug *P. solenopsis* and its parasitoid *A. arizonensis* were collected from cotton fields located in Ludhiana, Junagadh, Saoner and Chhindwara of India by random sampling method. Uniform infestation of mealybug and its parasitoid in cotton fields of major cotton-growing districts encouraged us to choose these sites for collection of the pest and the parasitoid. Details of the sampling locations are given in Table 1. Between 2000 and 2500 mealybugs were collected from cotton fields over a radius of 5 km in each location. The cotton mealybug *P. solenopsis* adult females bearing well-formed ovisacs were collected and brought to the laboratory. The mealybugs were then transferred to insecticide-free sprouted potato tubers *Solanum tuberosum* (L.) (Solanaceae). Mummified mealybug containing parasitoid *A. arizonensis* collected from these locations were brought to the laboratory and were reared on 3rd instar nymphs of *P. solenopsis* from the respective population. Laboratory populations of mealybug, *P. solenopsis* and its parasitoid *A. arizonensis* (unexposed to insecticides for at least 20 generations) were maintained as susceptible check for toxicity comparisons.

Matured adults of *P. solenopsis* and *A. arizonensis* were observed under the stereomicroscope and identified through morphological keys^54,55^. The insect populations were maintained in plastic cages (30 × 30 × 30 (L × B × H) containing sprouted potatoes for three generations before being used in the bioassays. Insect rearing was maintained at standardized environmental conditions, as follows: 25 ± 2 °C, 75 ± 5% R.H., 12L:12D photoperiod, according to the methodology developed by Nagrare et al.^56^. Non-Bt cotton plants, *Gossypium hirsutum* L, var. “LRA 5166” (Malvaceae) were grown from seeds under greenhouse conditions (20 ± 5 °C, 70 ± 10% R.H.) avoiding any pesticide application. The seeds of cotton variety “LR5166” were obtained from the ICAR Central Institute for Cotton Research, Regional Station, Coimbatore, Tamil Nadu, India. Clean cotton leaves used for the bioassays were collected from 60-day-old cotton plants that reached the phenological stage identified as BBCH Code 51^57^.

### Insecticide usage pattern in the study area

Knowledge Attitude Practice (KAP) surveys were conducted before the start of the experiment by following the protocol used by Yadouleton et al.^29^ and Naveen et al.^30^ to understand the insecticide usage pattern in the study area. Minimum of ten farmers in each locality were informally interviewed by using a semi-structured questionnaire focusing on insecticide usage patterns in each farm. Further, data were collected on cropping patterns and, control strategies through direct observations and group discussions. The details of the survey are presented in Table 1.

### Insecticides

Technical grades of imidacloprid (ai. 93%), thiodicarb (ai. 89%) (Sigma Aldrich, USA) and profenofos (ai. 89%) (Pesticide Industries Ltd., India) were used for the bioassays. Technical grade insecticides were dissolved in acetone, and serial concentrations were prepared using deionized water containing Triton X-100 (0.1 g L^−1^) as a non-ionic surfactant^30^. Based on the usage pattern of insecticides, these insecticides were selected, as they represented the OPs, pyrethroids and neonicotinoids concurrently used for control of mealybugs and other sucking pests in the respective regions where the mealybug populations were collected.

### Bioassays

#### Susceptibility levels of *P. solenopsis* populations

The level of susceptibility in *P. solenopsis* field populations to imidacloprid, profenofos and thiodicarb was assessed through the IRAC method 001 for toxicological bioassays with a slight modification from Nauen and Elbert^58^. Briefly, for each active ingredient, between 6 and 7 concentrations were prepared. Fresh cotton leaves previously infested with ten coetaneous 3rd instar nymphs (having the same age or date of origin) were dipped into the chosen insecticide concentration for 5 s and allowed to dry for 1 h under a fume hood in laboratory conditions. Once dried, each insecticide-sprayed cotton leaf was placed between two superposed ventilated Petri dishes (10 cm in diameter). The distal portion of the leaf petiole was immersed under a 2 ml Eppendorf^®^ tube filled with distilled water and sealed with Parafilm^®^. Mortality was recorded 24 h after exposure to insecticides. Mealybugs showing no coordinated movement or not responding when gently touched with a soft paintbrush were considered dead. Each insecticide-concentration combination and the control were replicated five times. This bioassay was conducted separately for each population of *P. solenopsis* under the above-mentioned experimental conditions.

#### Residual contact toxicity of insecticides on *A*. *arizonensis*

The residual contact toxicity of imidacloprid, profenofos and thiodicarb on each field population of *A. arizonensis* was evaluated through the method described by Desneux et al.^21^*.* Briefly, glass vials (12 × 5 cm) were filled with 2 ml of insecticidal solution, flipped horizontally and poured out for allowing them to dry. Thus, five couples (i.e., 5 females and 5 males) of newly emerged adult parasitoids (0–24 h-old) from the rearing were released into ventilated glass vials covered by a fine mesh net. Each insecticide for each concentration and the control were replicated five times. Mortality was assessed after 24 h. The parasitoids were considered dead if they did not respond when touched with a soft paintbrush. The bioassay for each population was conducted separately keeping the same laboratory conditions. Residual toxicity of these insecticides was compared with that of laboratory susceptible check for assessing relative tolerance of *A. arizonensis* field populations to insecticides.

#### Effect of insecticides on parasitization potential of *A. arizonensis*

The effect of imidacloprid, profenofos and thiodicarb on *A. arizonensis* was assessed by evaluating the reproductive traits (i.e., parasitism and emergence rates) of the survived adult females exposed to insecticide dry residues on glass. According to the methodology described above, 50 *A. arizonensis* mated females (24 h old) of each population were transferred from the rearing system into glass vials treated with the previously calculated median lethal concentration (LC_50_) of each insecticide. Glass vials treated only with a solution of acetone and water were included as control. Six hours after the exposure to insecticide residues on glass, 20 survived females were randomly selected and transferred in a glass jar containing a cotton leaf preliminary infested with a hundred 3rd instar nymphs of *P. solenopsis* from the respective population. Females of *A. arizonensis* were allowed to parasitize the mealybug nymphs for 24 h and then removed. The parasitism rate was recorded 7 days after the exposure by counting the number of mummified mealybugs that showed light brown color, while the parasitoid emergence rate was recorded after 12 days.

### Data analysis

The Levene and Shapiro–Wilk tests were used to check the homogeneity and normality of variance of the dependent variables and the dataset was log-transformed whenever needed. Mortality data from concentration–response bioassay were subjected to probit analyses^59^ using Polo Plus 2.0 software (LeOra Software, USA). The LC_50_ and LC_90_ values with Fiducial limits, slopes of the regression lines standard errors, and χ^2^ significance tests, were thus estimated. Values were considered significantly different whether their 95% fiducial limits did not overlap. The observed mortality was corrected for control mortality through Abbott’s formula. The parasitism rate was calculated as the per cent of parasitized mummies on the total offered mealybug hosts. The emergence rate was calculated as the per cent of emerged parasitoids on developed mummies. For assessing toxic effect of insecticide on the parasitoid, we tested the effect of insecticide, population and the potential interaction of these two factors (insecticide × *population*) on the proportion of developed mummies (i.e., parasitoid pupae) and the proportion of the newly emerged parasitoids by carrying out a one-way ANOVA followed by Least Significant Difference (LSD) post hoc test (P < 0.05) for multiple mean comparisons among the treatments. This analysis was performed in IBM^®^ SPSS^®^ Statistics for Macintosh, Version 23.0.0.0 (IBM Corp. Released 2015. Armonk, NY: IBM Corp).

### Likelihood of control failure of insecticides on field populations of *P. solenopsis*

The likelihood of control failure of insecticides was estimated based on Silva^60^ and Naveen et al.^30^. The current level of susceptibility of the field populations of *P. solenopsis* as given by the estimates of LC_50_ and 95% confidence limits for the tested insecticides was compared with the maximum recommended field dose of these insecticides by the Central Insecticides Board and Registration Committee (CIBRC), Government of India. As per CIBRC, the recommended doses for the tested insecticides against sucking pests were: imidacloprid 0.00625 mg L^−1^; profenofos: 0.125 mg L^−1^; thiodicarb: 0.185 mg L^−1^; The expected mortality of field populations of *P. solenopsis* as achieved by label rates in comparison with the estimated LC_50_ of the tested insecticides is represented graphically. The toxic impact of these insecticides on *A. arizonensis* field populations could be deduced from this graph.

### Environmental risk assessment (ERA)

The indirect effect of imidacloprid, profenofos and thiodicarb on parasitization potential of *A. arizonensis* was assessed by calculating the risk assessment parameters as described below.

#### Reduction coefficient (Ex)

The Ex, that summarizes the potential insecticide deleterious effects, was calculated as described by Urbaneja et al.^61^ using the formula:

$${\mathbf{E}}_{{\mathbf{x}}} = 100\left\{ {1 - \left[ {\left( {1 - {\text{E}}_{{{\text{mx}}}} {/}100} \right)\left( {1 - {\text{E}}_{{{\text{fx}}}} {/}100} \right)} \right]} \right\}$$

where E_mx_ represents the corrected mortality calculated as per Abbott^62^ of the parasitoid when exposed to a given insecticide, while, E_fx_ denotes parasitization capacity determined as follows:

$${\text{E}}_{{{\text{fx}}}} = 100 - \left( {{\text{F}}_{{\text{x}}} 100{\text{/F}}_{{\text{c}}} } \right)$$

where F_x_ and F_c_ represent the mean percent parasitization recorded for insecticide x and the untreated control, respectively. The Reduction coefficients (Ex) were used for classifying the insecticides according to the International Organization for Biological Control (IOBC) standards into four categories: (1) E_x_ < 30%—harmless; (2) E_x_: 30–80%—slightly harmful; (3) E_x_: 80–99%—moderately harmful; (4): E_x_ > 99%—harmful.

#### Descriptive analysis

Descriptive statistics E was calculated as described by Hassan et al.^27^

$${\text{E}} = \frac{{1 - {\text{T}}}}{{\text{C}}} \times 100$$

wherein E refers to the percent reduction in parasitism or emergence; T and C denote the mean percent reduction in parasitism or emergence in the treatment and control groups, respectively. Based on the E values range, the insecticides were grouped into four classes following the IOBC guidelines: (1) E < 30%: harmless (Class 1); (2) 30% ≤ E ≤ 79% as slightly harmful (Class 2); (3) 80% ≤ E ≤ 99% as moderately harmful (class 3); (4) E > 99% as harmful (class 4).

#### Risk index (RI)

The indirect toxic effect of insecticides on parasitoid *A. arizonensis* was expressed as Risk Index (RI) which refers to the reduction in natural potential parasitization due to insecticide application. Risk Index was calculated following Vercruysse and Steurbaut^63^.

$${\text{RI}} = \left( {{\text{RC}} - 25} \right){/}\left( {100 - 25} \right),\;{\text{with}}\;{\text{RC}}\;{\text{being}}\;{\text{the}}\;{\text{reduction}}\;{\text{in}}\;{\text{parasitization}}\;\left( \% \right).$$

#### Selectivity ratio

Selectivity ratio was estimated as described by Şengonca and Liu^64^ using the formula given below:

$${\text{Selectivity}}\;{\text{ratio}} = \frac{{{\text{LC}}_{50} \;{\text{of}}\;{\text{the}}\;{\text{parasitoid}}\;\left( {\upmu {\text{g}}\;{\text{ai}}\;{\text{L}}^{ - 1} } \right)}}{{{\text{LC}}_{50} \;{\text{of}}\;{\text{the}}\;{\text{pest}}\;\left( {\upmu {\text{g}}\;{\text{ai}}\;{\text{L}}^{ - 1} } \right)}}$$

Selectivity ratio < 1 indicates that the chemical is more toxic to the parasitoid than to the *P. solenopsis* (non-selective); The ratio > 1 indicates that the chemical is less toxic to the parasitoid.

#### Hazard quotient

The Hazard quotient^65^, was calculated to estimate the ecological risk of pesticides as follows:

$${\text{Hazard}}\;{\text{quotient}} = \frac{{{\text{Recommended}}\;{\text{field}}\;{\text{rate}}\;{\text{for}}\;P.\;solenopsis\left( {{\text{g}}\;{\text{ai}}\;{\text{ha}}^{ - 1} } \right)}}{{{\text{LC}}_{50} \;{\text{of}}\;A.\;arizonensis\left( {{\text{g}}\;{\text{ai}}\;{\text{L}}^{ - 1} } \right)}}$$

An hazard quotient < 50 indicates that the compound is non-hazardous to parasitoids for a given exposure rate.

### Ethics declarations

This study does not involve any human subjects.

## Data Availability

All the data were provided in the manuscripts.
